# A critical look into artificial intelligence and healthcare disparities

**DOI:** 10.3389/frai.2025.1545869

**Published:** 2025-03-06

**Authors:** Deborah M. Li, Shruti Parikh, Ana Costa

**Affiliations:** ^1^Renaissance School of Medicine, Stony Brook University, Stony Brook, NY, United States; ^2^Department of Anesthesiology, Renaissance School of Medicine, Stony Brook University, Stony Brook, NY, United States

**Keywords:** artificial intelligence, ethics, social determinants of health, SDH, health inequalities, technology in healthcare, social disparities in health

## Introduction

Artificial intelligence (AI) has permeated many aspects of daily life, including medicine, in recent years. As of 2021, 343 AI-enabled medical devices had been approved by the United States (US) Food and Drug Administration, with many more in development (Badal et al., 2023). Most notable thus far has been AI's ability to assist with every step of radiology workflow: it can determine the appropriateness of imaging, recommend the most appropriate imaging exam, predict wait times or appointment delays, and interpret imaging, with much more potential utilizations (Syed and Zoga, 2018). The World Health Organization proposed that AI tools be integrated into healthcare to improve efficiency and achieve sustainable health-related development (World Health Organization, 2021). AI in healthcare can reduce costs and administrative burdens, reduce waiting times for patients to receive care, improve diagnostic abilities and patient care, facilitate data management, and expedite discovery (Botha et al., 2024a,b).

However, the advancement of AI in healthcare comes with unique drawbacks. For example, data security and privacy are at risk and must be improved, as patients may more readily and unknowingly provide consent for covert data collection methods (Khan et al., 2023; He et al., 2019). Use of AI must be seriously reconsidered if it poses a risk to patient confidentiality, a non-negotiable in healthcare. With the ability of AI to rapidly gather and analyze large amounts of patient data, controlling the scope of its use becomes a challenge: these tools may progress to collect and disclose data without patient consent or direct investigator oversight (Botha et al., 2024a). In addition, as most healthcare-based AI research has been conducted in non-clinical settings, rolling out AI in certain clinical settings may result in non-evidence-based practice (Khan et al., 2023). For example, clinicians may feel tempted to use AI for tasks beyond their validation, and training data may not adequately represent the scenarios clinicians encounter (Nilsen et al., 2024). In fact, many studies on AI in healthcare have been administered in non-clinical settings (Botha et al., 2024a).

That is not to say AI should not be used in healthcare. It does, however, require immense consideration in how it is designed and why it is utilized. Some have contended that a goal of developing AI for healthcare should be to minimize health disparities and make the healthcare system more equitable (Badal et al., 2023; Campbell et al., 2021). Yet, many characteristics of AI make this goal difficult to achieve. As such, there is a growing body of literature that discusses AI's role in both closing and perpetuating these inequalities (Celi et al., 2022; d'Elia et al., 2022; Ali et al., 2023). As the ability of AI is directly proportional to the quality of the training sets used, authors have addressed concerns regarding bias in training datasets and lack of diversity in development teams ultimately resulting in AI-driven disparities in care (Botha et al., 2024a; Green et al., 2024; Ferrara, 2024; Haider et al., 2024).

This article draws from existing literature to add to the ongoing conversation about the implications of AI in healthcare disparities. Specifically, we discuss economic implications, the explainability of AI systems, and the importance of compassionate care. Ultimately, while AI may indeed confer benefits to the healthcare system, it remains far from the goal of closing healthcare disparities and may, instead, backfire.

## Economic implications

One essential consideration in any kind of social disparity is economics. The US is notorious for having the highest healthcare expenditure globally, with healthcare costing $3.5 trillion, or 17.9% of the Gross Domestic Product (Khanna et al., 2022). Any measure to decrease this economic burden—either in the US or internationally—may be attractive. AI has the potential to save billions in annual healthcare costs (Zhu et al., 2024). AI may greatly streamline workflow, even in non-clinical tasks. An automated system may alleviate administrative burdens such as scheduling patients, estimating wait times, and billing insurance companies (Syed and Zoga, 2018; Zhu et al., 2024; Knight et al., 2023). Such workflow optimization may reduce the cost of healthcare delivery by cutting out intermediaries that typically handle these mundane tasks. In turn, patients' financial responsibility related to their care may be reduced.

On the clinical side, AI may be used to screen for and diagnose conditions, stratify disease risk, and devise treatment plans (Khanna et al., 2022). It may significantly reduce medical errors and factors that are associated with adverse outcomes (Botha et al., 2024a). Eventually, as technology advances, it may even perform procedures, given that it is deemed ethical, safe and evidence-based. While these benefits may seem like simply a perk to those practicing in physician-rich areas, they could become indispensable to those in areas affected by shortages of medical professionals (Lamem et al., 2025). Urban and rural communities bear the brunt of this inequity, with many struggling to access both primary and specialty care (Kirch and Petelle, 2017). It has been estimated that by 2030, there may be a shortage of up to 104,900 physicians in the US (Kirch and Petelle, 2017). As such, AI implementation in these underserved populations may help to alleviate these challenges and improve disparities regarding access to care (Lamem et al., 2025). Furthermore, AI assistants may help decrease physician burnout and therefore improve quality of care (Lin et al., 2019).

These advantages of AI are conferred only with the proper development, installation and maintenance of these systems. AI requires immense investment. One model for an AI glaucoma screening tool in the Changjiang county in China estimated that the fifteen-year accumulated incremental cost of using this tool was $434,903.20 for ~2,000 patients (Xiao et al., 2021). While the costs of this screening tool are arguably worth early detection and reduced disease progression, it may be impractical to roll out to larger populations. Health institutions in wealthy countries may easily make this investment. But what about institutions in developing countries? Community hospitals with limited government funding? Practices in rural areas with less purchasing power? Even if analyses demonstrate that costs are saved in the long run, the upfront investment may be too large an obstacle (Khanna et al., 2022).

Once a system is developed, purchased, and installed, maintenance becomes another issue. Software updates, advanced computing technologies, and ever-increasing cloud storage requirements add to costs (Badal et al., 2023). The evolving cybersecurity needs to protect patient health information may create further barriers to widespread application of AI (Shah et al., 2023). These all-around cost barriers are more nuanced than the mere ability to implement AI in practice. Inevitably, there are AI algorithms with higher and lower levels of sophistication, infrastructures that are more and less robust, and security measures that are stronger and weaker. The AI system that institutions choose will be closely tied to their financial status. Of course, AI development will then leave behind under-resourced communities.

## The black box of AI

Currently, there is a lack of “explainable” AI regarding algorithms and data sets that play a role in decision making (Amann et al., 2020). In other words—exactly how do these technologies work? How are they making these decisions? These are questions that even developers themselves cannot answer; we know they work, yet nobody can fully explain how. This “black box” of AI holds important implications to healthcare disparities worldwide. Machine learning (ML) is a component of AI which involves automated decision making based on datasets (Tack, 2019). Detecting and correcting biases based on limited training sets is an ethical prerequisite of justice in AI- and ML-based clinical decision-making (Kirch and Petelle, 2017). In other words, explainable AI enables developers to identify and correct training set-based biases that currently skew algorithms (Celi et al., 2022; Green et al., 2024).

The discussion of justice behind explainable AI requires additional considerations. Explainable AI models keep model developers accountable for their work, as lack of accountability precedes error (Amann et al., 2020). This concern is compounded by the fact that patients who are less literate are less likely to ask questions or seek more information about their care (Katz et al., 2007). Since these patients may be less prepared to participate in shared decision making, they may not challenge questionable decisions (Keij et al., 2021).

AI should be treated as a tool to support decision-making, not one to make decisions independently. For example, AI prescription systems have been developed to aid physician workflow and prevent human error (Tantray et al., 2024; Tully, 2012). Inevitably, physicians will encounter scenarios in which the AI recommendation conflicts with their clinical judgement. Some of these scenarios may arise if AI systems are not trained on datasets that adequately represent the populations they treat, thereby generating recommendations poorly aligned with the realities of patients' needs (Botha et al., 2024a). This challenge is particularly relevant to minority communities that have historically been under-studied (Haider et al., 2024). Healthcare providers should critically assess the AI recommendation in the context of their clinical experience and patient preferences. Institutions should establish clear policies on how to accept or reject AI suggestions to maintain quality patient care.

Justice in explainable AI systems is important also in that more transparent technologies will foster patient trust in providers and the healthcare system. Unexplainable, opaque models, on the other hand, may exacerbate the mistrust that already pervades the healthcare system. This mistrust is particularly prevalent in socially and economically marginalized communities (Jaiswal and Halkitis, 2019). A key component of trust in underprivileged populations is the patient's comfort with the physician and physicians' personal involvement in patient care (Gopichandran and Chetlapalli, 2013). As such, we may see that the unexplainable black box of AI and ML – if not handled correctly – would certainly exacerbate these concerns. Lack of explanation for these impersonal, automated algorithms may further alienate this vulnerable population and widen health disparities.

## Compassionate care

Even if we are to elucidate the black box, can AI ever replace the physician-patient relationship in delivering empathic care? Currently, it seems unlikely – one recent study demonstrated that healthcare chatbots delivering both empathetic and sympathetic responses to patients in fact lowered patients' perception of their authenticity (Seitz, 2024). In contrast, empathy and sympathy expressed by human physicians did not induce this negative effect (Seitz, 2024). This lack of perceived authenticity may not only undermine patients' subjective satisfaction with their AI providers but may also objectively worsen patient outcomes. While some AI tools provided sound biomedical recommendations for diabetes management, they overlooked psychosocial components that are also necessary for glycemic control (Romero-Brufau et al., 2020). Algorithms that determine A1c goals, calculate medication dosages, and send prescriptions may certainly help optimize patient care. However, recommendations poorly tailored to psychosocial challenges disproportionately affect those with greater social barriers. Continuing the stand-alone case of diabetes, for example, significant social barriers to care include having the ability to afford healthy food, the free time for follow-up visits and the literacy to understand health information (Paduch et al., 2017). Now combine this diabetes with a slew of other health conditions, medications, unemployment concerns and an ailing family member. Surely, physicians can manage this patient in countless different ways. There is no one correct path. Regardless, it is imperative that health providers—human or AI-based—address these concerns with compassion.

Palliative care, which emphasizes relieving suffering and optimizing quality of life in end-of-life care, is a field in which compassion is key (Adegbesan et al., 2024). While AI may help assist in decision-making, it risks depersonalizing cases and lacking empathy when patients and their families need it the most. Death and dying are often rooted in culture, personal beliefs, and spirituality. The experience is deeply personal and unique to each family (Adegbesan et al., 2024). Whereas some encourage open communication about death, others feel uncomfortable with it; whereas some value life-prolonging measures regardless of prognosis, others less so (Ohr et al., 2017). Palliative care AI models risk imposing a “one-size-fits-all“ model of care based on a Western training dataset (Adegbesan et al., 2024). Once again, understudied populations and cultural minorities fall behind in AI's “understanding”—or lack thereof—of their values.

## Discussion

Society at large, including regulators, policy makers, insurance companies, healthcare professionals and patients should carefully consider incorporating AI practices into the practice and business of medicine. Regulators have raised concerns over the need for regulation of clinical AI as well as generalizability to different populations (Hogg et al., 2023). Another area of concern relevant to several stakeholders including healthcare providers and regulators is legal responsibility for AI clinical decision making (Hogg et al., 2023). This fear exists for physicians in the scenario of a medical error made by AI and conversely for accusations of negligent care for not using AI. Physicians were neither prepared for nor agreed with assuming responsibility for errors made by AI, while AI developers believed they should not be liable since they do not practice medicine (Hogg et al., 2023). Each side felt they only understood “part of the whole” when it comes to AI, further highlighting the need for explainable AI. Appropriate oversight by policy makers and regulators is needed to ensure accountability and promote development of explainable AI. These risks may be further mitigated by informing patients that AI was involved in decision making (Lorenzini et al., 2023). Certain narratives have pitted AI as a rival to the skills and education of physicians, with claims that AI will one day replace physicians (Lorenzini et al., 2023). AI remains solely a tool to assist in clinical setting with the final decision being made by a human. Rhetoric that continues to pit AI against physicians will only hinder the incorporation of AI into clinical practice (Lorenzini et al., 2023). Patients will not benefit from AI replacing their physicians, but they also will not benefit from avoiding AI altogether.

In discussing the role of AI in closing healthcare disparities, we must consider the role of AI in low- and middle-income countries (LMICs). In areas where medical resources and personnel are scarce, AI can reduce the workload on healthcare personnel (Schwalbe and Wahl, 2020). AI can also improve access to medical care, especially for areas where specialty care is not available (López et al., 2022). Disease outbreaks can be predicted earlier and allow for mobilization of treatment to affected areas. ML has been used to assess disease severity and predict treatment failure for illnesses such as malaria, tuberculosis and dengue fever (Schwalbe and Wahl, 2020). However, LMICs face significant challenges in implementing AI. The lack of electronic health records and health data is a limiting factor since this data is the primary input used in AI algorithms (López et al., 2022). Most AI systems are developed in high income countries (HICs) and the ML models reflect datasets from those populations. When applying these technologies to LMICs, models must be updated to reflect the population it is being applied to. Failure to do so can reinforce and exacerbate existing health disparities (López et al., 2022).

Gaining both physician and patient trust in the integration of AI in healthcare remains a problem that needs to be addressed in the future. In a small study interviewing patients on their perspectives on AI in general medical practice, subjects had mixed feelings on implementation of AI (Mikkelsen et al., 2023). A common concern amongst participants related to sharing of and access to their medical information (Mikkelsen et al., 2023). The patients wanted assurance that appropriate consent would be obtained prior to sharing of their data and that anonymization would be used. A survey of 203 participants on public opinion of AI in medicine also yielded mixed results, with a near 50/50 split when asked if they trust AI as a physician's tool for diagnosing medical conditions (Rojahn et al., 2023). In the same study, a majority of participants trusted a human physician over AI in making a culturally biased decision. There was a more positive outlook toward the future as over 25% of respondents believe AI will improve medical treatment in the next 10 years and nearly half of respondents for the next 50 years (Rojahn et al., 2023).

Similarly, what AI lacks that physicians have is not intelligence, but rather wisdom—the sense of intuition that a human being can accumulate only over time (Powell, 2019). Can AI develop this intuition over time? Can an AI model mimic the human brain in synthesizing decades' worth of information to analyze a unique case and provide appropriate medical decision making? For simple cases, it likely can. But complex cases are a different story—risks and benefits of intervention must be weighed and complications must be predicted, all while delivering this information to the patient in an easily-digestible manner. Yet another layer of nuance is added when shared-decision making is introduced—now, AI must understand patients' desires and uncertainties on a human level and incorporate that into its recommendations. Moreover, patients believe their physician should remain the primary decision maker, with AI can be used as a support tool (Mikkelsen et al., 2023). The use of AI in this setting may decrease time physicians spend on mundane tasks and leave more time for physicians to have meaningful conversations with their patients, facilitating the delivery of compassionate care (Hogg et al., 2023; Sauerbrei et al., 2023). Once again, compassion and trust are key components to patient care for all patients alike.

## Conclusion

While AI has the potential to increase access to care for vulnerable populations and help bridge gaps in healthcare, we must ensure that data and algorithms are inclusive of patients to avoid worsening existing disparities. The healthcare system hinges on trust to maintain patient confidentiality, recommend the optimal course of action, and execute the plan appropriately. Particularly in marginalized communities, the critical process of building and maintaining this trust has proved difficult even in the absence of AI and continues to pose a significant obstacle in the success of AI to improve healthcare delivery. Both physicians and patients alike do not wish to see AI replace the standard physician-patient interaction. Instead, AI can serve as an adjunct to improve quality of care by reducing the chance of human error. Collaboration among patients, physicians, and AI developers is essential to achieve this goal in an equitable manner.
